# 3A-Amino-3A-Deoxy-(2AS, 3AS)-β-Cyclodextrin Hydrate/Tin Disulfide Modified Screen-Printed Carbon Electrode for the Electrochemical Detection of Polychlorinated Biphenyls

**DOI:** 10.1186/s11671-019-3236-z

**Published:** 2020-01-03

**Authors:** Xinke Liu, Rajalakshmi Sakthivel, Chia-Yin Cheng, Jiangliu Luo, Lijun Song, Jianhua Wu, Wei He, Usman Younis, Ren-Jei Chung

**Affiliations:** 1 College of Materials Science and Engineering, Chinese Engineering and Research Institute of Microelectronics, Shenzhen University, Shenzhen, 518060 People’s Republic of China; 2 Department of Chemical Engineering and Biotechnology, National Taipei University of Technology (Taipei Tech), Taipei, 10608 Taiwan; 3 Research Center of Guangdong Intelligent Charging and System Integration Engineering Technology, Shenzhen Winsemi Microelectronics Co., LTD., Shenzhen, 518000 People’s Republic of China; 4 College of Electronics and Information Engineering, Shenzhen University, Shenzhen, 518060 People’s Republic of China

**Keywords:** Polychlorinated biphenyl, 3A-amino-3A-deoxy-(2AS,3AS)-β-cyclodextrin, Tin disulfide, Disposable screen-printed carbon electrode, Cyclic voltammetry

## Abstract

Polychlorinated biphenyls (PCBs) are persistent organic pollutants that are widely distributed in the environment. It is noteworthy that the PCBs are endocrine-disrupting substances, and their toxicity induces cancer and damage to the mammalian reproductive system, immune system, stomach, skin, liver, etc. This work aimed to synthesize 3A-amino-3A-deoxy-(2AS, 3AS)-β-cyclodextrin hydrate/tin disulfide composite material and to study its material properties, electrochemical properties, and application to PCB detection. The nanostructured tin disulfide (SnS_2_) synthesized by hydrothermal technique and 3A-amino-3A-deoxy-(2AS, 3AS)-β-cyclodextrin hydrate were sequentially modified onto the disposable screen-printed carbon electrode (SPCE) via titration using a micropipette. The 3A-amino-3A-deoxy-(2AS, 3AS)-β-cyclodextrin hydrate (β-CD) improved the selectivity of the modified electrode. The fabricated β-CD/SnS_2_/SPCE was employed to determine the presence of PCBs by cyclic voltammetry (CV) and differential pulse voltammetry (DPV). The detection range was 0.625–80 μM, with a limit detection of approximately 5 μM. The electrodes were as stable as 88% after 7 days’ storage. The results showed that the β-CD successfully encapsulated PCBs to achieve an electrochemical sensor that reduced the time and increased the convenience of PCBs detection.

## Introduction

Recently, studies on the removal of persistent organic pollutants (POPs) from the environment and protecting the global environment are significant [1]. Polychlorinated biphenyls (PCBs) are ubiquitous pollutants that are widely spread in the environment [2] and extensively applied in different branches of industry, owing to their excellent chemical properties, physical properties [3], lack of combustibility, thermal stability, and dielectric properties. In addition, PCBs are broadly employed in various industries as insulating fluids and coolants in electrical tools in power plants and huge buildings [4–6]. Since the 1970s, the production and commercial use of PCBs has been prohibited in some countries because of their bioaccumulation, environmental persistence, and strong toxicity [1]. However, excessive PCBs are found in various products, such as heat-conducting liquids and capacitors [3]. The trade name of the PCB mixture studied is Aroclor, which is manufactured by Monsanto Chemical Company in the USA. Further, the Aroclor PCB mixture contains over 100 diverse specific PCBs congeners. Conversely, the frequent use of PCBs can create some problems worldwide in the soil, aquatic environments, and air, and even in the human body [7, 8]. Moreover, the persistent nature of PCBs in the environment can induce negative health effects in human and animals. Therefore, the enhancement of PCB-detection methods is extremely important in the global environment. Today, traditional methods like liquid chromatography-mass spectrometry (LC/MS) and gas chromatography-mass spectrometry (GC/MS) [9–11] are used to detect PCBs. Nevertheless, these methods have some disadvantages, namely the need for qualified personnel, high cost, high time consumption, and difficulty and complexity of sample preparation [12, 13]. Hence, quantity control of PCBs requires low-cost, rapid techniques, and an on-site analysis system. Electrochemical methods have been used in different potential applications and environmental investigation for their advantages, such as easy miniaturization, simple instrumentation, good quantitative determination, rapid response time, and high selectivity and sensitivity. Till date, only a limited number of articles have been reported to be based on the electrochemical determination of PCBs [14]. Further, the unmodified electrode has a low electron-transfer rate and poor conductivity. Therefore, the modification with nanostructured or different types of materials is significant. As a result, the 3A-amino-3A-deoxy-(2AS,3AS)-β-cyclodextrin with tin disulfide was employed for the fabrication on screen-printed carbon electrode (SPCE) (β-CD/SnS_2_/SPCE).

**Fig. 1:**
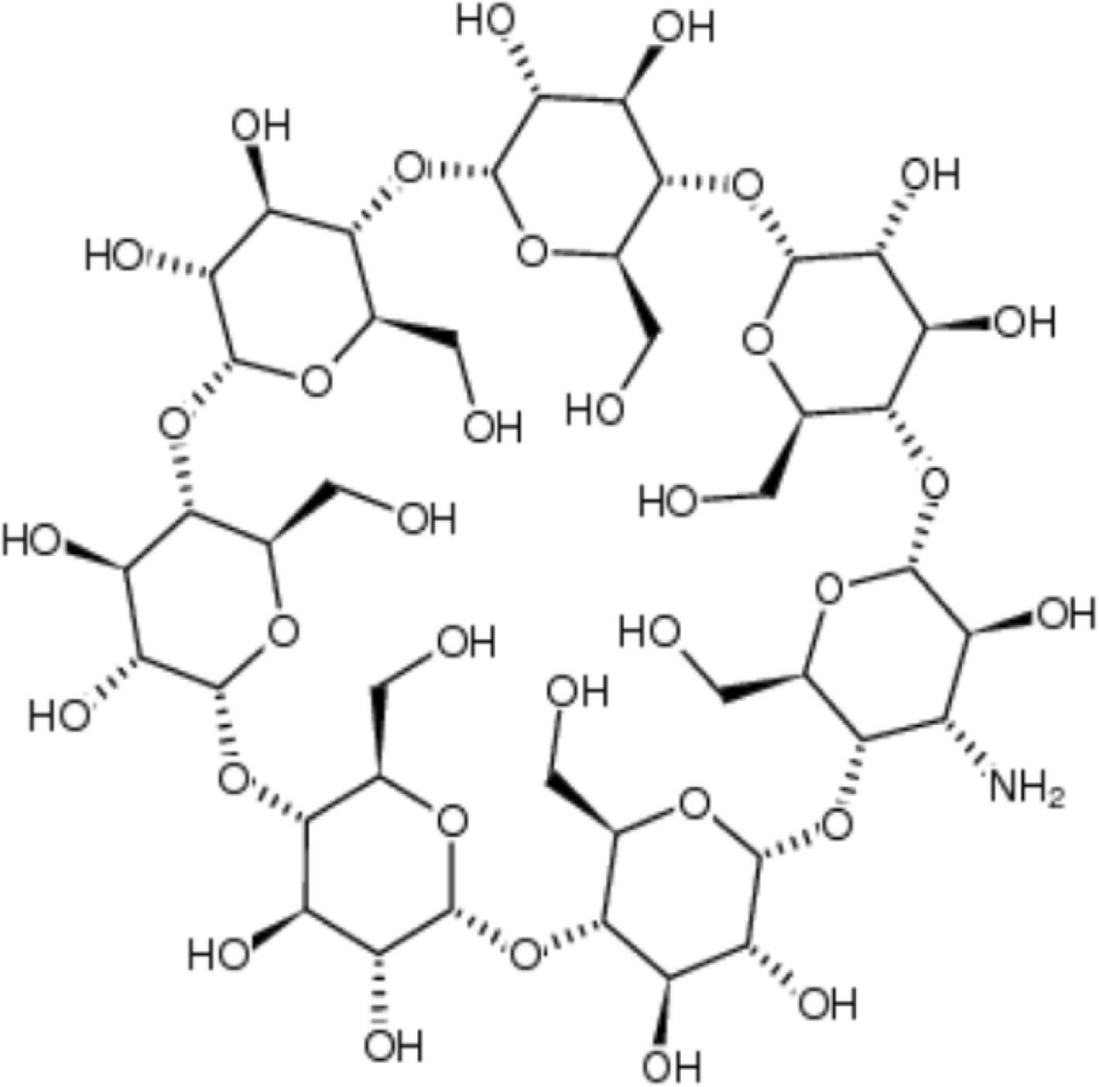
The structure of 3A-amino-3A-deoxy-(2AS,3AS)-β-CD

Cyclodextrin (CD) is a common term for cyclic oligosaccharides, which are classified from five or more glucopyranose molecules. Five monomers polymerized CD does not occur in nature. Generally, the natural CDs are classified as α-CD, β-CD, and γ-CD, which are composed of six, seven, and eight glucopyranose units. The CD has a hydrophilic characteristic in the outer ring and hydrophobic characteristic inside the ring of the molecule. It has a stereoscopic conical cavity of a certain size and encapsulates the molecules in the benzene rings [15]. This special molecular hole structure allows the CD cavity to combine with a weakly polar compound or functional group to form a host-guest interaction. Then, the hydrophilic outer wall of the CD enhances the water solubility. Furthermore, β-CDs are the most commonly used molecules, owing to their low-cost production and moderate cavity size [16]. In recent years, the CD has been extensively applied in the pharmaceuticals, food, and chemical industries as well as agriculture and environmental engineering. In this work, the CD is used in the form of 3A-amino-3A-deoxy-(2AS,3AS)-β-CD hydrate, and its structure is shown in Fig. 1.

Tin disulfide (SnS_2_) is one of the member of IV–VI metal dichalcogenides (MDCs) which is an important n-type semi-conductor with the indirect band gap of 2.2 eV [17]. The SnS_2_ has developed as an important building block for their sustainable electronic and optoelectronic applications. The SnS_2_ has a layered cadmium diiodide (CdI_2_) crystalline like structure comprising the sandwiched tin atom between two sulfur atoms (S-Sn-S) with covalent bonds, and the adjacent sulfur layers are connected to each other through van der Waals attraction [18]. The SnS_2_ material has been widely utilized in research, owing to their potential applications including optoelectronics, nanoelectronics, light harvesting, and energy-conversion applications [19]. Furthermore, the maximum theoretical activity of the SnS_2_ nanomaterial exhibits better compatibility and applicability in the electrochemical sensor [20]. As a result, the SnS_2_ nanomaterial was used for the preparation of β-CD/SnS_2_ composite.

In this study, we demonstrate the synthesis of SnS_2_ and the preparation of β-CD/SnS_2_ composite material. The SnS_2_ nanomaterial was synthesized through the hydrothermal synthesis method. The 3A-amino-3A-deoxy-(2AS,3AS)-β-CD hydrate were sequentially modified onto disposable screen-printed carbon electrode (SPCE) by titration using a micropipette. 3A-amino-3A-deoxy-(2AS,3AS)-β-CD hydrate (β-CD) improved the selectivity of the modified electrode. The resultant material was probed by favorable spectrophotometric and voltammetric techniques. The fabricated β-CD/SnS_2_/SPCE was used for the electrochemical detection of PCBs.

## Materials and Methods

### Materials

Thioacetamide (C_2_H_5_NS, 98%) and tin tetrachloride pentahydrate (SnCl_4_·5H_2_O, tetrachlorostannane) were purchased from Alfa (USA) and Showa (Japan). Methanol (CH_3_OH, methyl alcohol 99.9%) obtained from J.T. Baker. Disodium hydrogen phosphate (Na_2_HPO_4,_ sec-sodium phosphate ≥ 99%), sodium dihydrogen phosphate (NaH_2_PO_4_, monosodium phosphate ≥ 98%), sodium hydroxide (NaOH, caustic soda ≥ 97%), potassium hexacyanoferrate(II) ((K_4_[Fe(CN)_6_]), potassium ferrocyanide 98.5–102.0%), and potassium hexacyanoferrate(III) ((K_3_[Fe(CN)_6_]), potassium ferricyanide < 10 μm, 99%) were received from Sigma-Aldrich, Germany. The 3A-Amino-3A-deoxy-(2AS,3AS)-β-CD (C_42_H_71_NO_34_.XH_2_O, DTXSID20462166) was bought from basechem (http://www.basechem.org) and PCBs (Aroclor 1016) (C_12_H_7_C_l3_, certified reference material, 200 μg/mL in methanol) was received from Merck, Sigma Aldrich (Germany).

### Instruments

The surface morphological property of the synthesized material are investigated using field-emission scanning electron microscopy (high-quality imaging and advanced analytical microscopy (FE-SEM ZEISS (Sigma, Germany)). The crystalline nature of the two-dimensional (2D) hexagonal SnS_2_ materials was probed by X-Ray powder diffraction (XRD) and the XRD data were collected through the X’Pert3 Powder (PANalytical/Nederland). A powder diffraction analysis yields an X-ray diffractograms, exhibits the phase concentration (peak areas), crystalline phases present (peak position), crystalline size/strain (peak widths), and amorphous content (background hump). The pH tester pH 510 (Eutech Instrument/UK) was used to monitor the pH in the entire experiment. The electrochemical features and electrode kinetics of various modified electrodes were tested using CHI6114E, CH Instruments/USA. When the conventional three-electrodes were used, the SPCE is served as a working electrode, an Ag/AgCl and Pt electrodes were served as a reference and counter electrode. The electrolyte contains a mixed solution of 3 mM yellow blood salt (K_4_[Fe(CN)_6_]), 3 mM red blood salt (K_3_[Fe(CN)_6_]), and 0.1 M potassium chloride (KCl) solution. The scanning range of applied potential window is − 0.6 V–1.0 V and the scanning rate is 0.05 V/s.

### Synthesis of Tin Disulfide

Initially, approximately 0.351 g of tin precursor SnCl_4_·5H_2_O and 0.3 g of C_2_H_5_NS were mixed with 70 mL of deionized water. The solution mixture was stirred for 1 h in the room temperature. Then, 1 M NaOH was slowly added to adjust the pH of the solution and maintained the solution pH about 10.5. Later, the well-dispersed homogeneous solution mixture was poured into a stainless steel hydrothermal autoclave and heated in an oven from at 25 to 200 °C (first stage heating: 25 °C → 200 °C, 1 h; second stage heating: 200 °C, 11 h). After heating, the solution was cooled to room temperature. Then, the collected solution was washed several times by centrifugation using deionized water and ethanol (6000 rpm, 30 min). Finally, the tin disulfide powder was dissolved in deionized water, poured it into an evaporating dish, and dried in an incubator.

### Preparation and Fabrication of β-CD/SnS_2_ with Modified SPCE

**Fig. 2:**
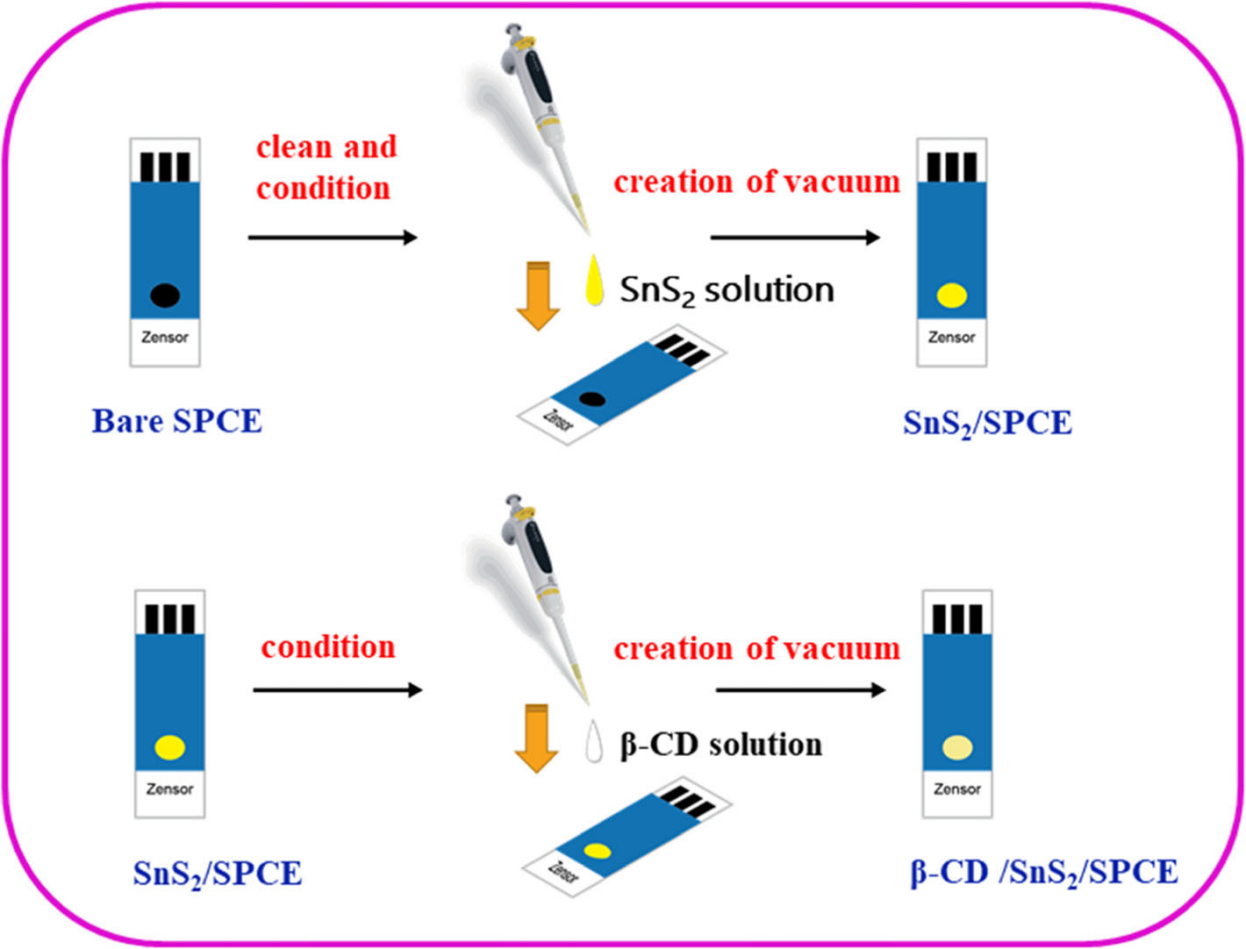
The preparation and fabrication of β-CD/SnS_2_/SPCE

First, 1 mM β-CD solution was prepared in 100 mL of deionized water. On the other hand, 0.02 g of SnS_2_ dissolved in 5 mL of deionized water and titrate 2 μL of the SnS_2_ solution with a micropipette onto the surface of the SPCE. Then, it was dried in a vacuum dryer for 10 min and titrated dry five times. Afterwards, the 2 μL of the aqueous solution containing β-CD is titrated on the surface of the nano SnS_2_-modified SPCE and dried for 10 min in a vacuum dryer. The prepared β-CD/SnS_2_ material modified with SPCE and the fabrication of β-CD/SnS_2_/SPCE are shown in Fig. 2.

## Results and Discussion

### Crystal Structure Analysis of SnS_2_

**Fig. 3:**
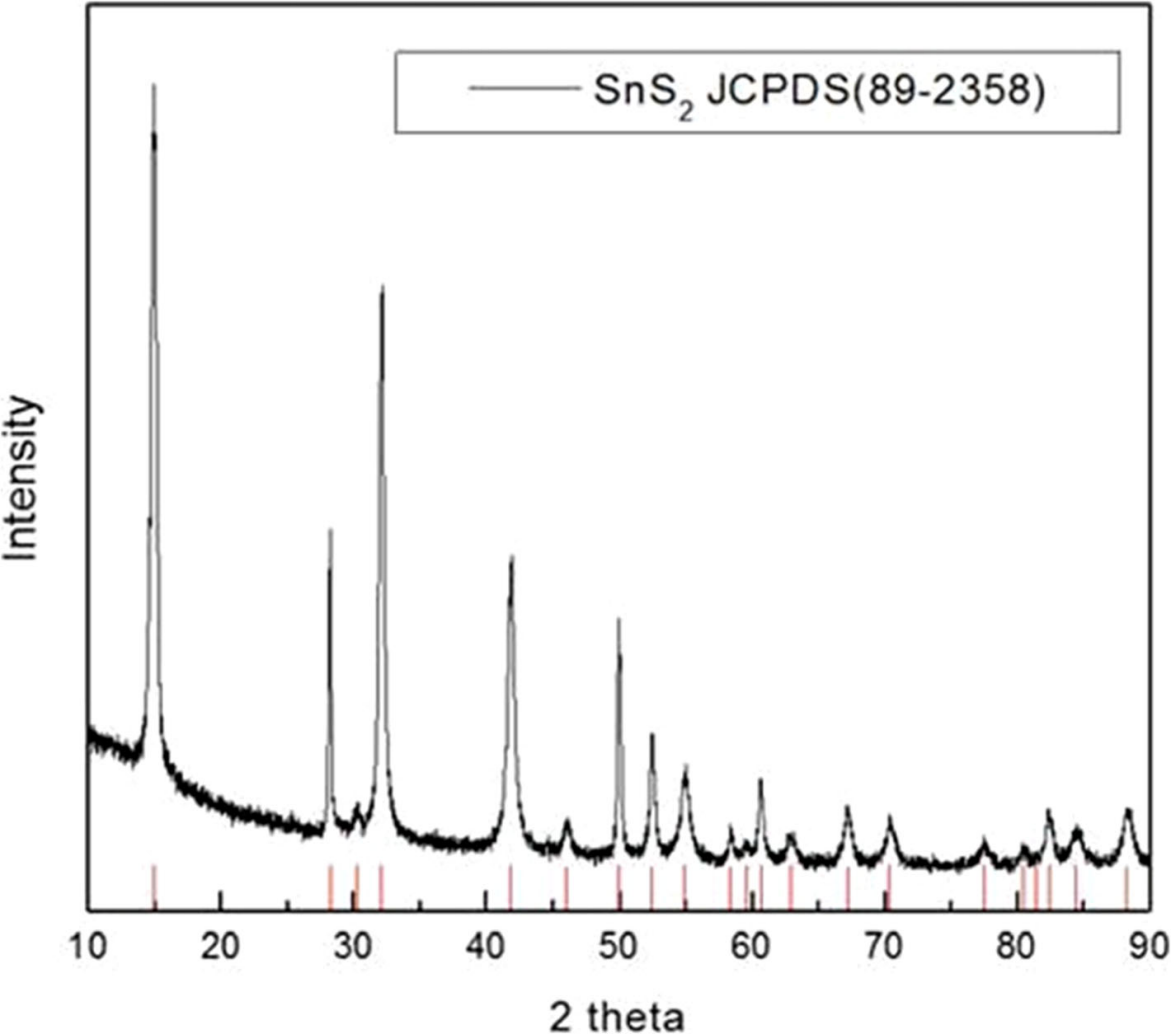
The XRD pattern of SnS_2_

The crystalline nature of the as-synthesized SnS_2_ was evaluated using XRD. Figure 3 shows the diffraction pattern of SnS_2_ exhibited the peaks at 15°, 29°, 30°, 31°, 41°, 46°, 50°, 51°, 53°, and 70°, which are attributed to the (001), (100), (011), (002), (012), (003), (110), (111), (103), and (113) planes, respectively. These results showed the hexagonal phase of SnS_2_ [JCPDS (89-2358)], which is confirmation of SnS_2_ formation [21].

### Surface Morphological Analysis of SnS_2_

**Fig. 4:**
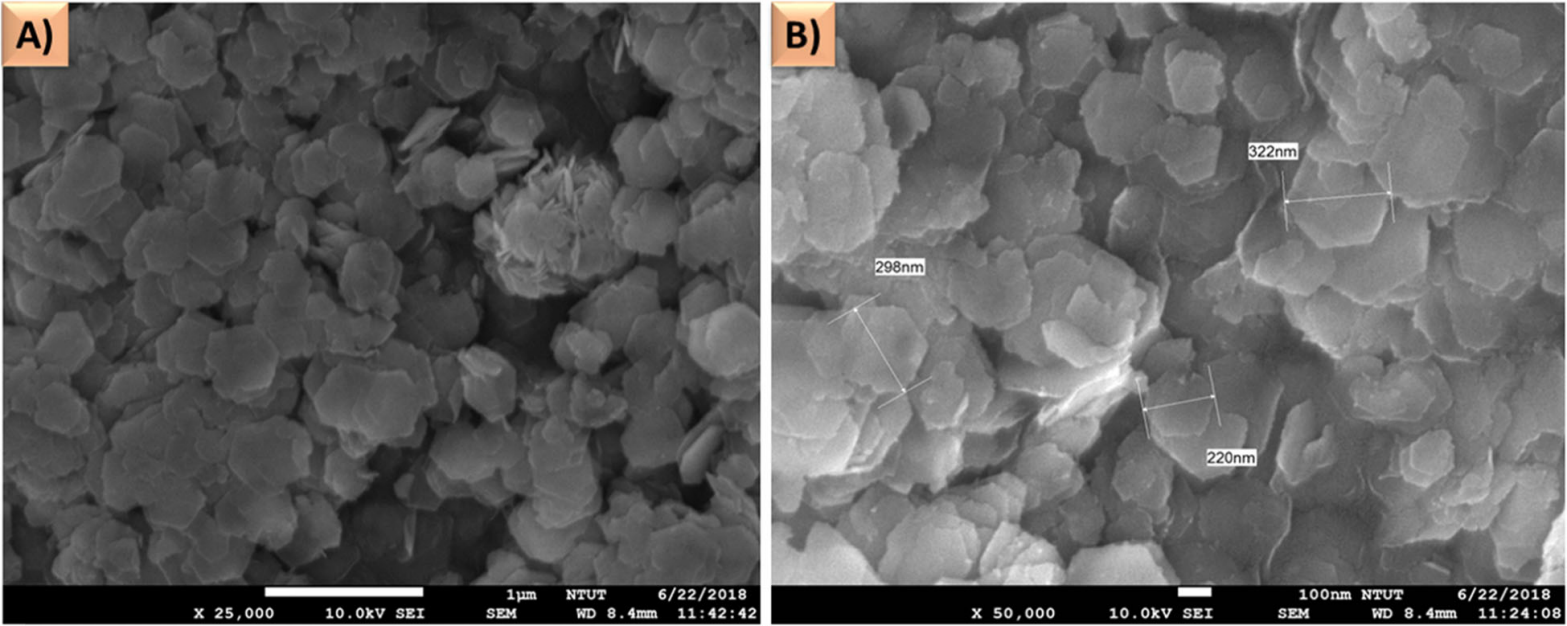
**a** The FESEM images of SnS at different magnifications. **b** The nanoflakes have widths of approximate 322, 298, and 220 nm

The surface morphology of the SnS_2_ material was examined using FE-SEM, and the images are shown in Fig. 4. It can be seen that the nanoflake like structured SnS_2_ with the hexagonal shape. At higher magnifications (Fig. 4a) and (Fig. 4b), the SnS_2_ has widths of approximate 322, 298, and 220 nm.

### Electrochemical Impedance Analysis and Effect of Electrolyte Solution

**Fig. 5:**
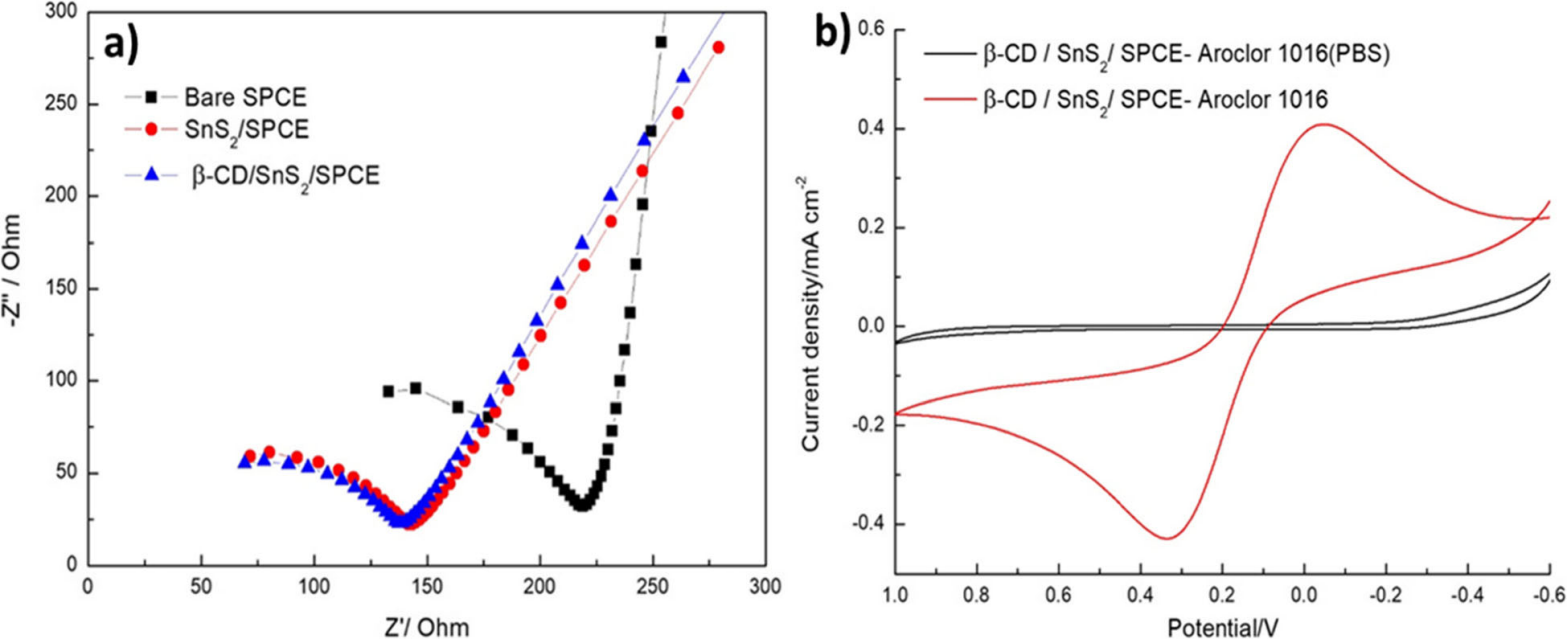
**a** EIS spectra of different modified electrodes: bare SPCE, SnS_2_/SPCE, and β-CD/SnS_2_/SPCE. **b** β-CD/SnS_2_/SPCE modified electrode in PBS (pH = 7.4) (black) and 3 mM yellow blood salt, 3 mM red blood salt, 0.1 M KCl solution (red) in the mixture solution of 80 μM PCB Aroclor (1016)

The electrochemical impedance analysis was tested on three different modified electrodes like bare SPCE, SnS_2_/SPCE, and β-CD/SnS_2_/SPCE and the results are shown in Fig. 5a. It can be seen that the bare SPCE shows a large semicircle region and higher charge transfer resistance because the bare SPCE got the lower conductivity. Then, SnS_2_-modified SPCE has a lowest charge transfer resistance than the bare SPCE due to material modification of SPCE. Furthermore, the β-CD/SnS_2_/SPCE exhibits the fast electron-transfer rate and high conductivity than other electrodes. Hence, the fabricated β-CD/SnS_2_/SPCE is employed for the further electrochemical application.

The working electrode β-CD/SnS_2_/SPCE was tested in two different types of electrolytes: electrolyte (1): 10 mM concentration of phosphate-buffered saline (PBS), pH = 7.4; and electrolyte (2): 3 mM yellow blood salt, 3 mM red blood salt, 0.1 M potassium chloride (KCl). These two electrolyte solutions containing mixture of 80 μM PCBs (Aroclor 1016) were scanned by cyclic voltammetry (CV) at the applied potential voltage of − 0.6–1.0 V and scan rate of 0.05 V/s. It can be seen from Fig. 5b, the peak shape of the electrolyte 1: PBS electrolyte is not noticeable. In comparison, the electrolyte (2) exhibited a well-defined redox peak with maximum peak current response. Therefore, the electrolyte (2) is favorable for the detection of PCBs (Aroclor 1016).

### Electrochemical Performances of Different Modified Electrodes

**Fig. 6:**
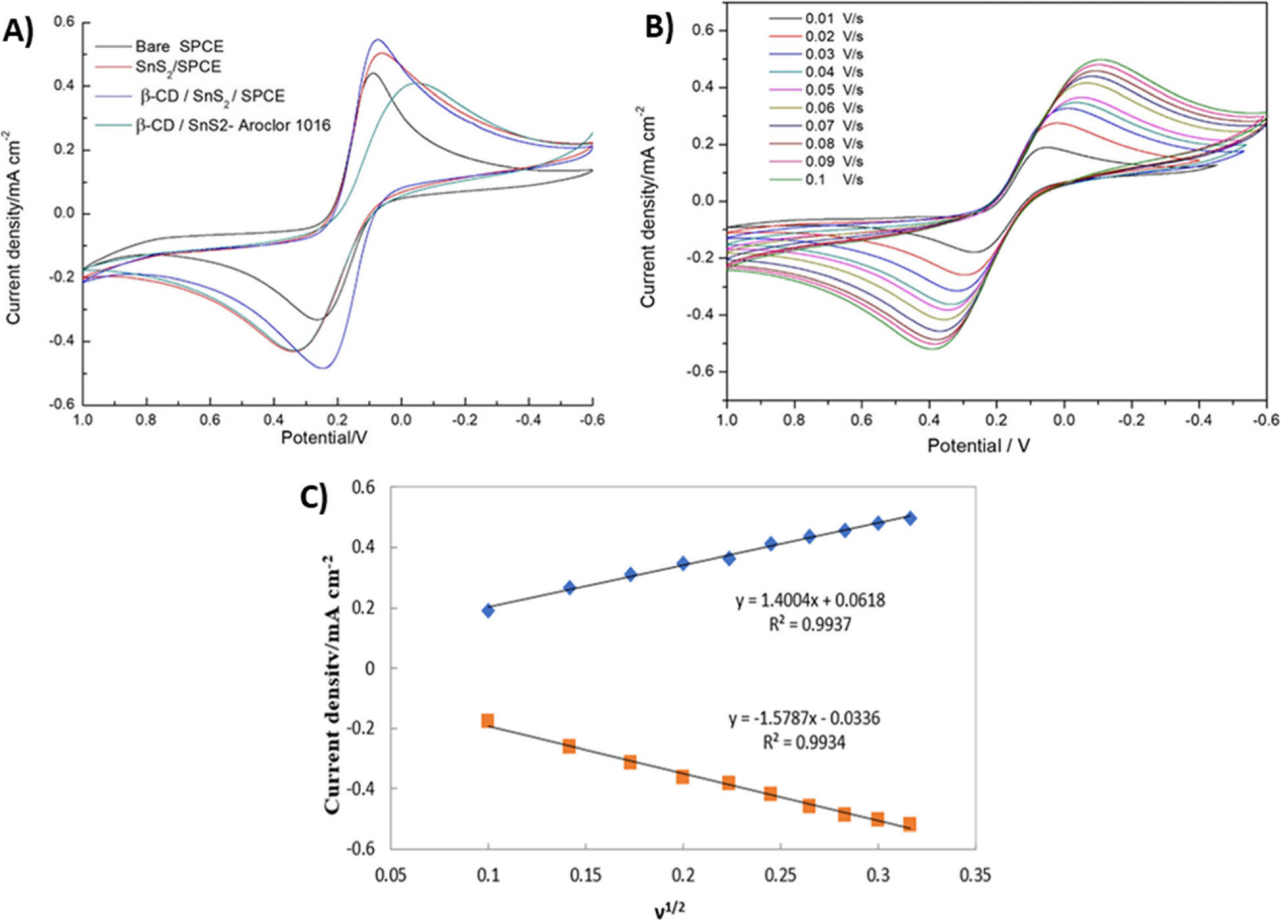
**a** CV curves of the first three electrodes: bare SPCE, SnS_2_/SPCE, and β-CD/SnS_2_/SPCE in the electrolyte containing a mixture of 3 mM yellow blood salt, 3 mM red blood salt, 0.1 M KCl solution, and other β-CD/SnS_2_/SPCE in an electrolyte containing PCBs (Aroclor 1016) potential window from − 0.6 to 1.0 V with a scanning rate of 0.05 V/s. **b** CVs of different scan-rate analysis (0.01 V/s to 0.1 V/s) was carried out in 80 μM PCBs (Aroclor 1016) in mixed solution of 3 mM yellow blood salt, 3 mM red blood salt, and 0.1 M KCl. **c** The calibration plot depicts the square root of the scan rate versus current density of the anodic and cathodic peak

The electrochemical performance of various modified electrodes, namely bare SPCE, SnS_2_/SPCE, and β-CD/SnS_2_/SPCE, was investigated using cyclic voltammetry (CV). The first three electrodes (bare SPCE, SnS_2_/SPCE, and β-CD/SnS_2_/SPCE) immersed in the electrolyte contains a mixture of 3 mM yellow blood salt and 3 mM red blood salt in 0.1 M KCl solution and the potential window from − 0.6 to 1.0 V, scanning rate at 0.05 V/s. Further, the β-CD/SnS_2_/SPCE was immersed in an electrolyte containing PCBs (Aroclor 1016) and recorded with the same procedure. As shown in Fig. 6a, the SnS_2_/SPCE has a significant current enhancement compared to bare SPCE. β-CD/SnS_2_/SPCE displays a higher current than other modified electrodes, owing to its good conductivity and does not hinder electron transfer. Finally, the β-CD/SnS_2_/SPCE was immersed in the electrolyte containing PCBs (Aroclor 1016) solution, and the current density suddenly decreased. Because of the hydrophobic cavity of β-CD was combined with PCB molecule and the host–guest interaction between the electrode surface β-CD and PCB. Then, the substances hinder the redox ([Fe(CN)_6_]^3−/4−^) molecule from reaching the electrode surface and which hinders the electrochemical process. When PCBs enter the cavity of the CD, there is a significant drop in conductivity.

### Effect of Scan Rate

The analyses were performed at different scan rates to check the reaction kinetics and investigate the influence on peak current and potential. The β-CD/SnS_2_/SPCE was used as the working electrode, and the electrolyte was a mixed solution of 3 mM yellow blood salt, 3 mM red blood salt, and 0.1 M potassium chloride (KCl). Then, 80 μM PCBs (Aroclor 1016) was added to the mixed solution and was scanned by CV. The experiment was conducted with different scanning speeds ranging from 0.01 to 0.10 V/s. As can be seen from Fig. 6b, when increasing the scanning rates, the electrochemical reaction time becomes shorter and the current response is increased. Conversely, when the scanning rate is smaller, the electrochemical reaction time is longer and the current response is smaller. As shown in Fig. 6b, the peak current values are linearly regressed by the square root of different scan rates (V^1/2^), whereas the redox peak current (Ipa and Ipc) were linearly proportional to the square root of scan rate. These results demonstrate that the electrochemical reaction process is a diffusion-controlled process. Further, the correlation coefficient value of anodic and cathodic peak was realized at *R*^2^ = 0.9937 and *R*^2^ = 0.9934 (Fig. 6c). Further, the electron-transfer rate constant value (*k*_s_) was calculated based on the Laviron equation [22].

1 $$ \log {\mathrm{k}}_{\mathrm{s}}=\upalpha \mathrm{log}\left(1-\upalpha \right)+\left(1-\upalpha \right)\log \upalpha -\log \left(\frac{\mathrm{RT}}{\mathrm{nF}\upupsilon}\right)-\frac{\left(1-\upalpha \right)\upalpha \mathrm{nF}\Delta  {\mathrm{E}}_{\mathrm{P}}}{2.3\mathrm{RT}} $$

Where *k*_s_ is the electron-transfer rate constant, α is the charge transfer coefficient, *n* is the electron-transfer coefficient of the molecule during the reaction, ν is the scan rate, *A* is the electrode surface area, *R* is the gas constant, *F* is the Faraday constant, *T* is the temperature, and ΔEp is peak potential difference.

The following equation is used to determine the value of α:

2 $$ {E}_{\mathrm{P}}-{E}_{\mathrm{P}/2}=\frac{0.048}{\upalpha \mathrm{n}} $$

Herein, *E*_p/2_ is a half-peak potential and other parameters are similar. The values are α = 0.236, *n* = 1, ν = 0.05 (V/s), *A* = 0.071 (cm^2^), *R* = 8.314 (J K^− 1^ mol^− 1^), *F* = 96,485 (C mol^− 1^), *T* = 298 (K), and ΔEp = 0.39(V).

After calculation, the electron-transfer rate constant ks = 0.039(s^−1^) can be obtained. In addition, the surface coverage value was calculated by the different scan rate analysis using the following equation: [23].

3 $$ {I}_{\mathrm{P}}=\frac{{\mathrm{n}}^2{\mathrm{F}}^2\mathrm{A}\uptau \upupsilon}{4\mathrm{RT}} $$

Where τ is the surface coverage and *I*p is the anodic peak current; the other parameters have already been explained. *I*_P_ =2.702 × 10^−5^ (A) and *n* = 1, and all other values are the same as those in the previous equation. The value of surface coverage (τ) was then found to be 0.814 × 10^−8^ mol cm^−2^.

### Effect of Different Concentration

**Fig. 7:**
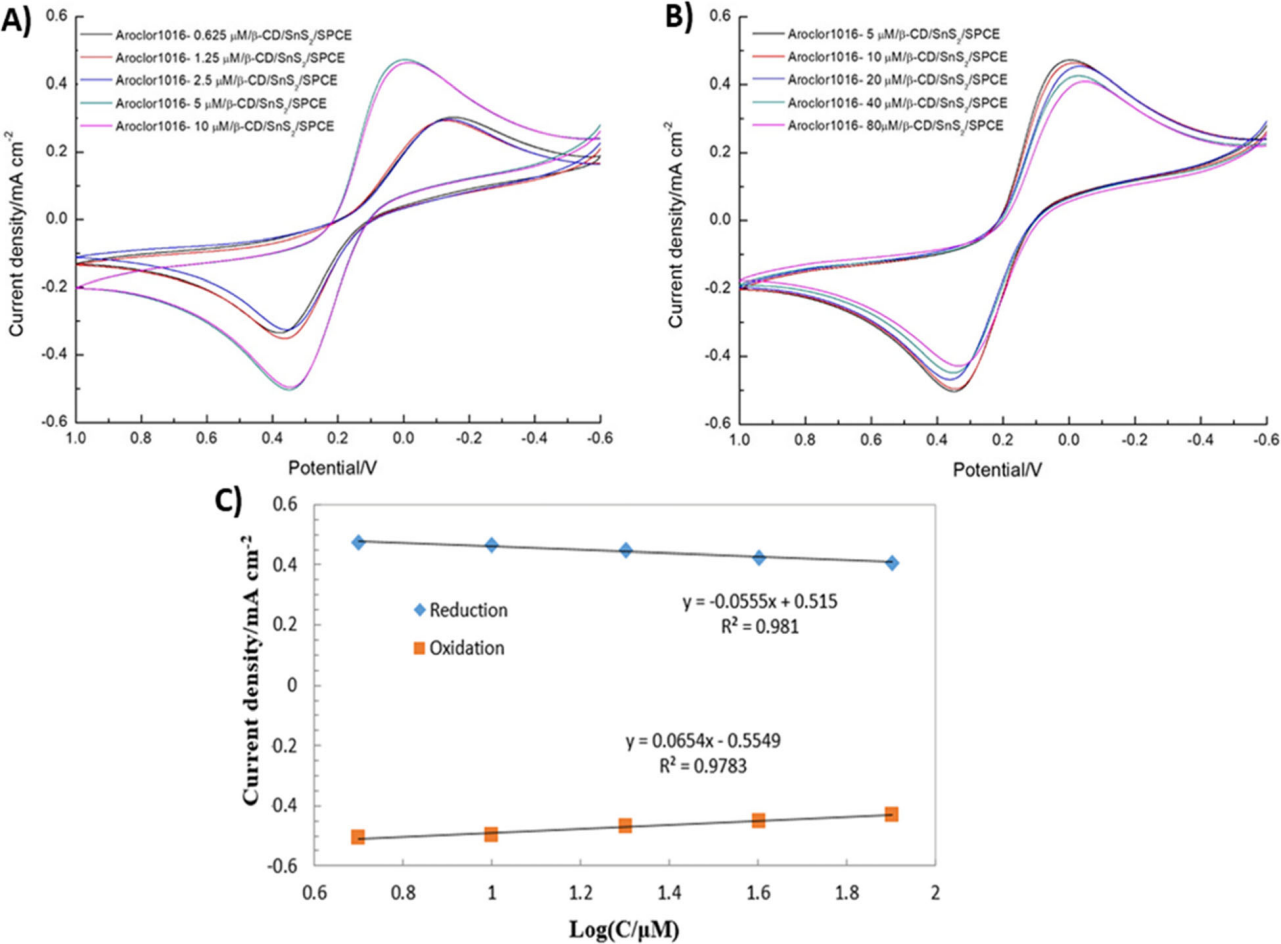
CVs of the β-CD/SnS_2_/SPCE at **a** different concentrations of added PCBs (Aroclor 1016) from 0.625 to 2.5 μM, **b** different concentrations of added PCBs (Aroclor 1016) from 5 μM to 80 μM. **c** The plot between the log concentration of PCBs (Aroclor 1016) and the anodic and cathodic peak current density

The electrocatalytic activity of β-CD/SnS_2_/SPCE at different concentration addition of PCBs (Aroclor 1016) was evaluated using CV. Figure 7a shows the CV curves of PCBs (Aroclor1016) and there were no changes between the concentration of 0.625 and 2.5 μM. The significant changes of CV were obtained only after the addition of 5 μM PCBs (Aroclor 1016) or more. Figure 7b shows the CV curves according to the PCBs (Aroclor 1016) concentrations of 5, 10, 20, 40, and 80 μM. It can be observed that when the concentration of PCBs (Aroclor 1016) increased, the redox reaction of [Fe(CN)_6_]^3−/4−^ was inhibited. The molecular diffusion reaches the surface of the electrode, which hinders the electrochemical process. The resistance of the electron transfer is proportional to the number of molecules of the CD-trapped PCBs (Aroclor 1016). Hence, the measured current signal intensity was gradually decreased by addition of PCBs (Aroclor 1016). These results demonstrate that the current detection limit of PCBs (Aroclor 1016) is 5 μM. Moreover, Fig. 7c displays that the redox current measured from the concentration of PCBs (Aroclor 1016) 5–80 μM had a linear relationship with the logarithm of the concentration. The resulting correlation coefficient *R*^2^ values of oxidation and reduction are 0.9783 and 0.981, respectively. This demonstrates that the β-CD/SnS_2_/SPCE achieved excellent electrocatalytic activity.

### Differential Pulse Voltammetry Analysis

**Fig. 8:**
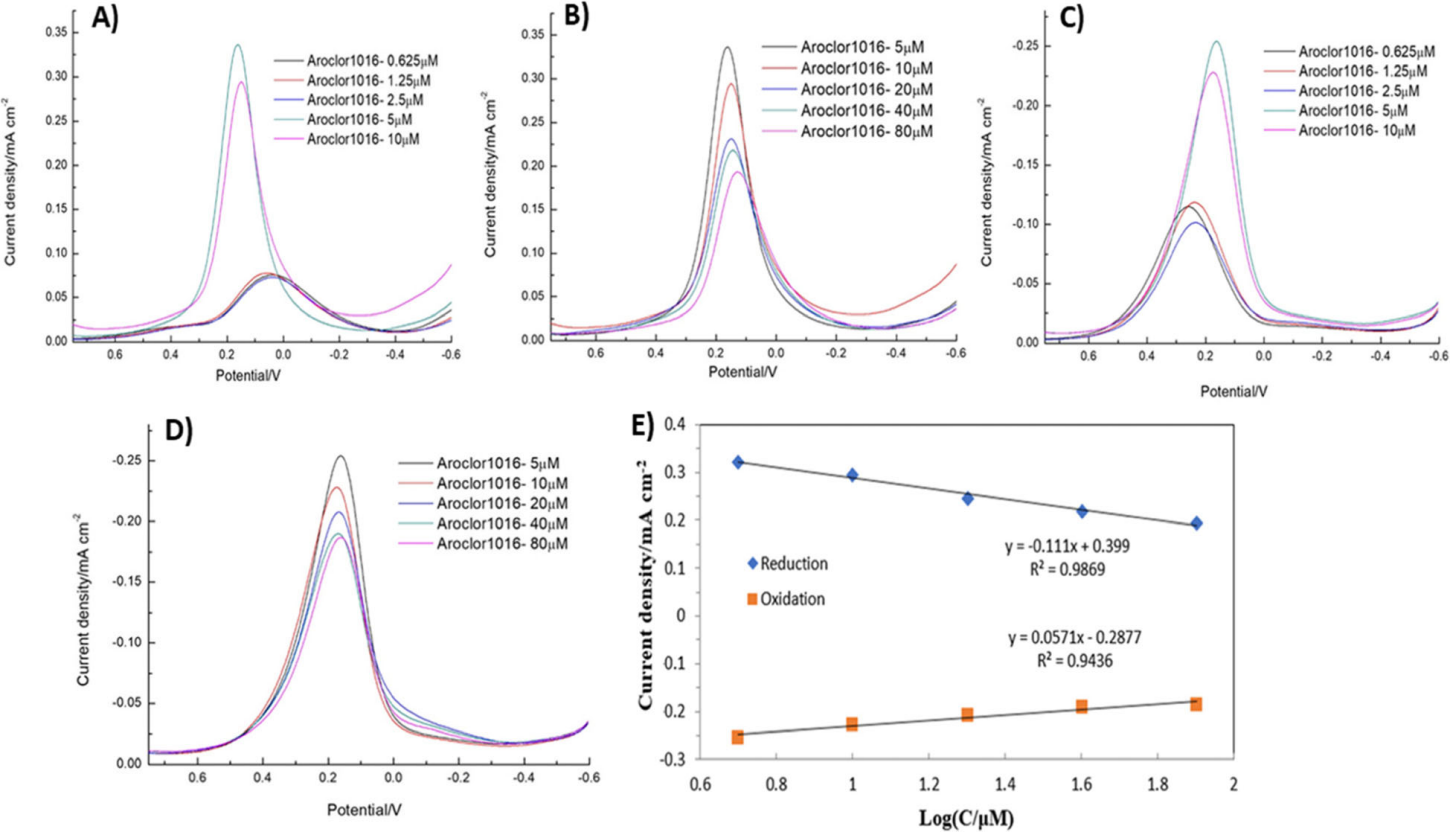
**a**, **b** DPV response of the reduction peak current depends on the different concentration addition of PCBs (Aroclor 1016). The different concentration addition of PCBs (Aroclor1016) at 0.625–10 μM into the electrolyte solution (**a**). The higher concentration addition of PCBs (Aroclor 1016) (5–80 μM) (**b**). **c**, **d** The oxidation peak current depends on the different concentration addition of PCBs (Aroclor 1016). **e** The plot between the oxidation and reduction peak current density versus log concentration of PCBs (Aroclor 1016)

**Fig. 9:**
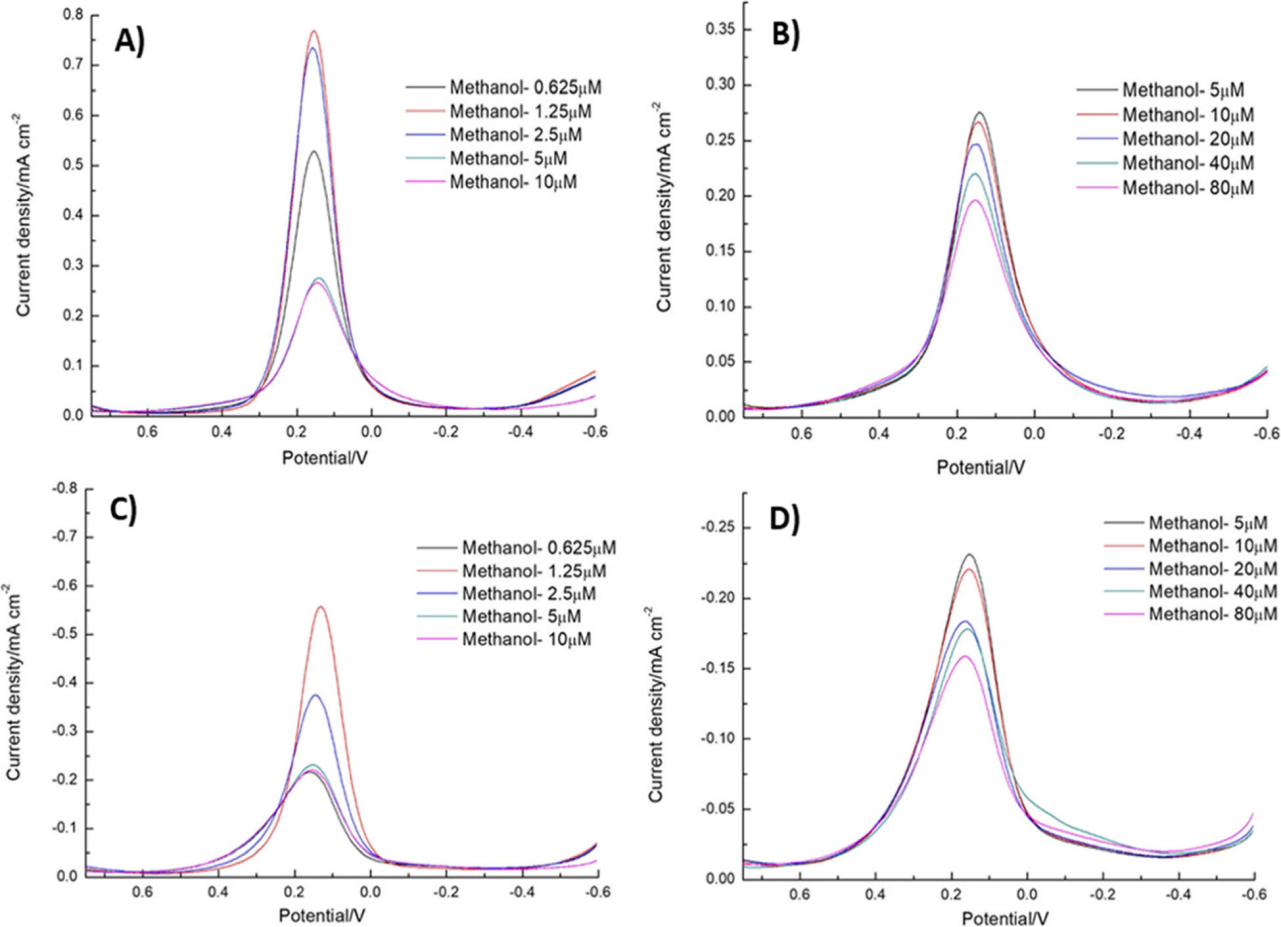
**a**, **c** Displays the reduction and oxidation peak current depends on the concentration of PCBs 1.25–10 μM dissolved in electrolyte methanol. **b**, **d** Exhibits the highest concentration addition of PCBs (Aroclor 1016) (5 to 80 μM) into the electrolyte methanol and corresponding the reduction and oxidation peak current

The differential pulse voltammetry (DPV) analysis method is a highly sensitive method compared to other voltammetric techniques. The various concentrations—0.625 μM, 1.25 μM, 2.5 μM, 5 μM, 10 μM, 20 μM, 40 μM, and 80 μM—of PCBs (Aroclor 1016) were measured by DPV method (Fig. 8a–d). Then, the control groups with methanol concentrations of 0.625 μM, 1.25 μM, and 2.5 μM were tested. The samples with concentrations of 5 μM, 10 μM, 20 μM, 40 μM, and 80 μM were tested separately (Fig. 9a–e). Figure 8a and b exhibit the reduction peak current depends on the different concentration addition of PCBs (Aroclor 1016). Figure 8a shows the different concentration addition of PCBs (Aroclor1016) at 0.625–10 μM into the electrolyte solution. The current intensity was gradually increased up to 5 μM, after addition the current was suddenly decreased. Figure 8b shows the higher concentration addition of PCBs (Aroclor 1016) (5–80 μM); the current density was linearly decreased. Because the PCBs are encapsulated in the β-CD cavity as a hydrophobic guest molecule. When the guest inclusion forms, the redox of [Fe(CN)_6_]^3−/4−^ is blocked because [Fe(CN)_6_]^3−/4−^ is not able to reach the electrode surface, and this phenomenon hinders the electrochemical reaction process. When the PCBs enter the hydrophobic cavity of the β-CD, the current signal strength dropped. As the similar experiment of DPV in Fig. 8c, d, but here mentioned the oxidation peak current of PCBs (Aroclor 1016). In Fig. 8e, the linear regression for the reduction reaction was *y* = − 0.111x + 0.399 with the correlation coefficient (*R*^2^ = 0.9869) and that of the oxidation reaction was *y* = 0.0571x − 0.2877 with *R*^2^ = 0.9436; these values are obtained from Fig. 8b, d. The electrochemical determination of PCBs based on β-CD/SnS_2_/SPCE compared with previous reports and the results listed in Table 1.

**Table 1:** The electrochemical performance of PCBs based on SnS_2_/β-CD/SPCE compared with previous reports

Modified electrode	Linear range (nM/L)	LOD (nM/L)	Ref
Antibody-immobilize on conducting polymer	1.1 to 3.8 × 10^2^	^a^1.3 × 10^1^, ^b^5.3, ^c^1.2, and ^d^6.4	[24]
Screen-printed electrochemical immunosensor	0.1 to 10	7 × 10^−3^	[25]
Functionalized magnetic beads and carbon based screen-printed carbon electrodes	–	^a^1.1, ^b^1.4, ^c^1.5, and ^d^3.1	[26]
Food samples (sheep milk, bovine adipose tissue, and bovine muscle)	^f^3.6 × 10^1^ to 1.8 × 10^5^	^e^3.6 × 10^1^	[2]
Antibody coated magnetic beads	–	^e^3.6 × 10^1^ and ^f^3.9	[27]
Label-free impedimetric immunoassay	^h^3.3 × 10^−2^ 3.3 × 10^1^	^g^3.3 × 10^−3^	[28]
Direct competitive enzyme-linked immunosorbent assay	^d^3.1 × 10^−2^ to 4.6	^c^3 × 10^−2^	[29]
Silver nanoparticles doped polyaniline modified glassy carbon electrode	^g^7.8 × 10^−1^ and 4.6	^f^2.4 × 10^−1^	[30]
SnS_2_/β-CD modified screen-printed electrode	625 to 8 × 10^4^	5 × 10^3^	This work

^a^PCB concentration expressed as Aroclor 1242

^b^PCB concentration expressed as Aroclor 1248

^c^PCB concentration expressed as Aroclor 1254

^d^PCB concentration expressed as Aroclor 1016

^e^PCB concentration expressed as Aroclors 1242 and 1248

^f^PCB concentration expressed as PCB28

^g^PCB concentration expressed as Aroclors of 1242, 1248, 1254, and 1260; 1:1:1:1

**Fig. 10:**
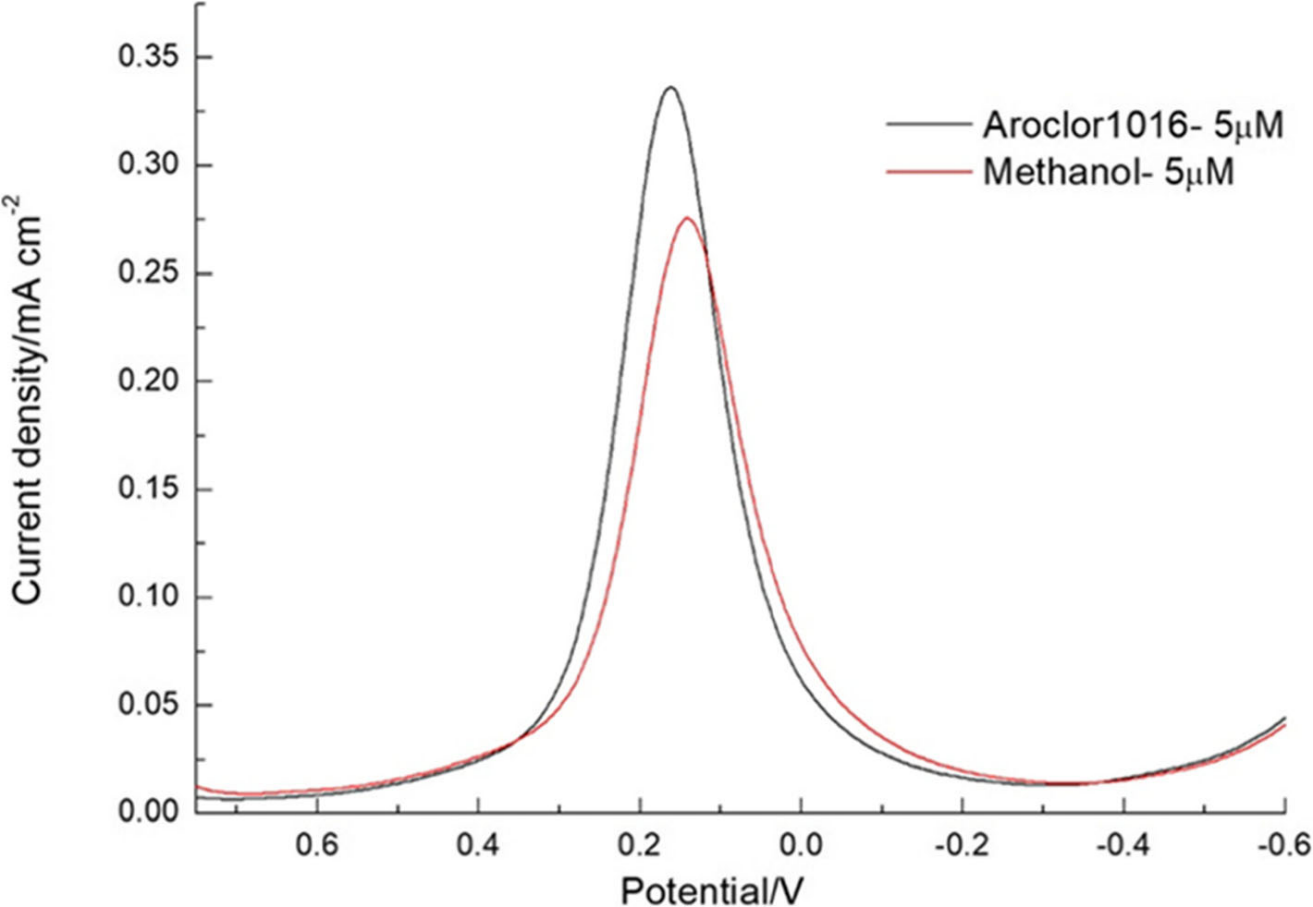
DPV response for the comparison of 5 μM added PCBs (Aroclor 1016) in methanol with the methanol-only solution

Furthermore, Fig. 9a, c displays that the reduction and oxidation peak current depends on the concentration of PCBs (Aroclor 1016) 1.25–10 μM dissolved in electrolyte methanol. From Fig. 9a, c, the maximum current was obtained at the concentration of 1.25 μM then the current response was decreased for higher addition. Furthermore, Fig. 9b, d shows the highest concentration addition of PCBs (Aroclor 1016) (5 to 80 μM) into the electrolyte methanol and corresponding the reduction and oxidation peak current. Whereas, when increasing the concentration of PCBs (Aroclor 1016), the current linearly decreased. Owing to the inclusion complex formation of PCBs between β-CD. Moreover, Fig. 10 shows the comparison of 5 μM concentration of PCBs (Aroclor 1016) in methanol and without methanol. The higher reduction current was obtained for PCBs (Aroclor 1016) without the addition of methanol. This result explains that the lowest detection limit of Aroclor1016 is 5 μM and methanol is 1.25 μM. The β-CD/SnS_2_/SPCE detects the analyte PCBs (Aroclor 1016), although it contains methanol. However, that is not affected by methanol implying that β-CD is combined with PCBs (Aroclor 1016). The affinity is higher than that of methanol, and the β-CD forms a host–guest inclusion complex through the hydrophobic cavity encapsulated PCBs (Aroclor 1016).

### Stability Test

**Fig. 11:**
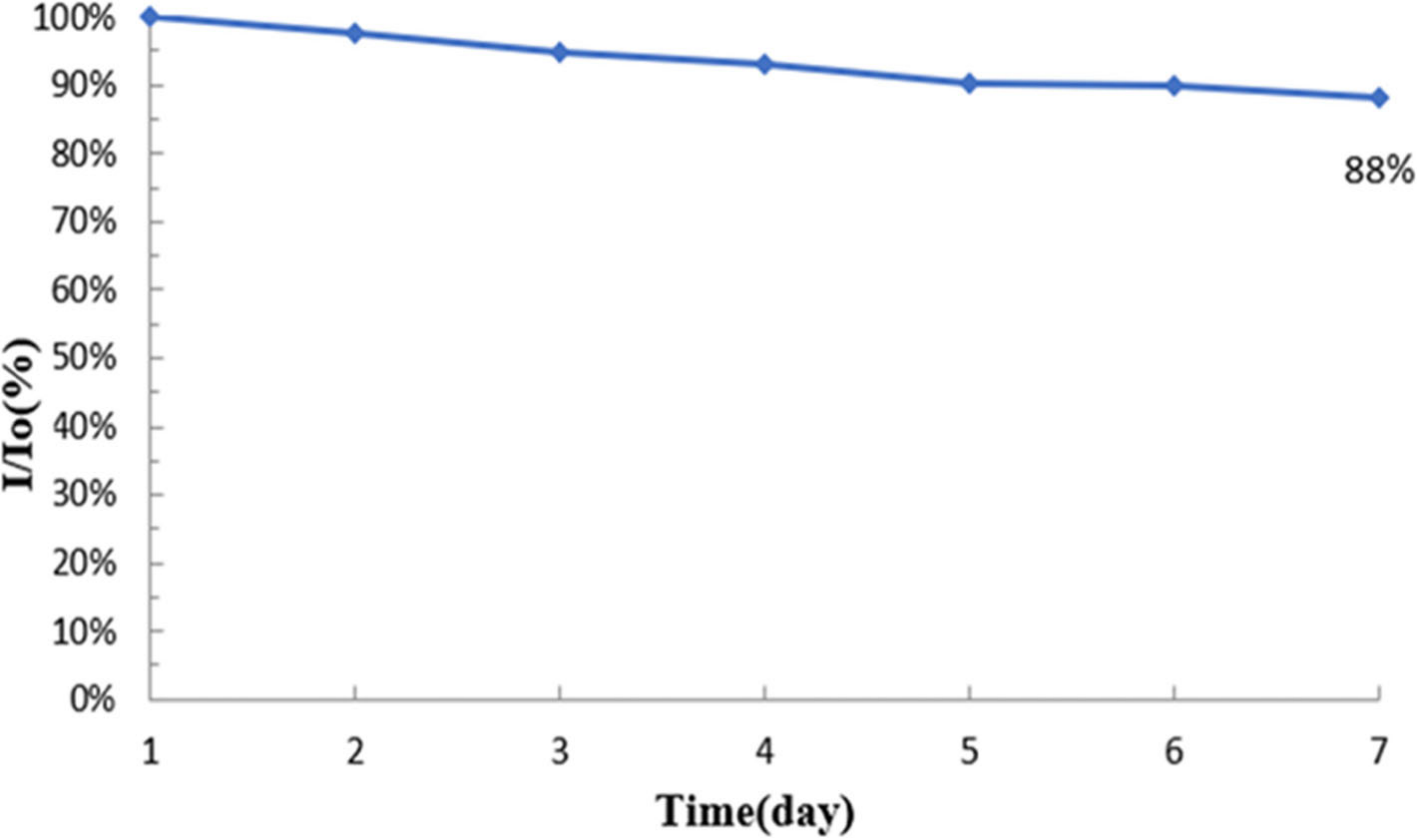
Stability test chart placed at room temperature for 7 days on β-CD/SnS_2_/SPCE

The stability of the β-CD/SnS_2_/SPCE was investigated by CV. The stability study experiments were performed for 7 days and the working electrode was stored at room temperature. The current change was measured once a day; here, the initial day current value is *I*_0_ and the change of current value is *I*. The current variation was calculated using the division of each day’s current value by the initial current value; the corresponding data plot is shown in Fig. 11. It can be seen that the β-CD/SnS_2_/SPCE displays a stability value to 88% at room temperature (7 days).

## Conclusion

In this manuscript, we demonstrated the hydrothermal synthesis of nano-tin disulfide (SnS_2_). The β-CD/SnS_2_/SPCE was fabricated using titration method by micropipette. The fabricated β-CD/SnS_2_/SPCE was successfully applied for the determination of PCBs (Aroclor 1016). Interestingly, the modified electrode has a linear detection range from 0.62 to 80 μM and a detection limit of 5 μM. Furthermore, the electrodes were as stable as 88% after 7 days’ storage. The results showed that the β-CD successfully encapsulated PCBs to achieve an electrochemical sensor that reduced the time and increased the convenience of PCBs detection. The fabricated modified electrode exhibits a rapid, facile, and sensitivity to electrochemical detection of PCBs. The proposed PCB sensor, the hydrophobic cavity of β-CD was connected with PCB molecule and the host–guest interaction between the electrode surface β-CD and PCB. The significant PCB electrochemical sensor shows a wide linear range, stability, sensitivity, reduced working time, and good reproducibility.

## Data Availability

All data generated or analyzed during this study are included in this published article.

## Abbreviations

2D: Two-dimensional
CV: Cyclic voltammetry
DPV: Differential pulse voltammetry
EIS: Electrochemical impedance spectroscopy
FE-SEM: Field emission scanning electron microscope
GC/MS: Gas chromatography-mass spectrometry
LC/MS: Liquid chromatography-mass spectrometry
MDCs: Metal dichalcogenides
PBS: Phosphate-buffered saline
PCBs: Polychlorinated biphenyls
POPs: Persistent organic pollutants
SnS_2_: Tin sulfide
SPCE: Screen-printed carbon electrode
XRD: X-ray diffraction
β-CD: 3A-Amino-3A-deoxy-(2AS, 3AS)-β-cyclodextrin hydrate
